# Substrate and Inhibitor Specificity of the Type II p21-Activated Kinase, PAK6

**DOI:** 10.1371/journal.pone.0077818

**Published:** 2013-10-28

**Authors:** Jia Gao, Byung Hak Ha, Hua Jane Lou, Elizabeth M. Morse, Rong Zhang, David A. Calderwood, Benjamin E. Turk, Titus J. Boggon

**Affiliations:** 1 State Key Laboratory for Conservation and Utilization of Subtropical Agro-biosciences, The Key Laboratory of Ministry of Education for Microbial and Plant Genetic Engineering, and College of Life Science and Technology, Guangxi University, Nanning, Guangxi, China; 2 Department of Pharmacology, Yale University School of Medicine, New Haven, Connecticut, United States of America; 3 Department of Cell Biology, Yale University School of Medicine, New Haven, Connecticut, United States of America; 4 Yale Cancer Center, Yale University School of Medicine, New Haven, Connecticut, United States of America; Institute of Molecular and Cell Biology (IMCB), Singapore

## Abstract

The p21-activated kinases (PAKs) are important effectors of Rho-family small GTPases. The PAK family consists of two groups, type I and type II, which have different modes of regulation and signaling. PAK6, a type II PAK, influences behavior and locomotor function in mice and has an ascribed role in androgen receptor signaling. Here we show that PAK6 has a peptide substrate specificity very similar to the other type II PAKs, PAK4 and PAK5 (PAK7). We find that PAK6 catalytic activity is inhibited by a peptide corresponding to its N-terminal pseudosubstrate. Introduction of a melanoma-associated mutation, P52L, into this peptide reduces pseudosubstrate autoinhibition of PAK6, and increases phosphorylation of its substrate PACSIN1 (Syndapin I) in cells. Finally we determine two co-crystal structures of PAK6 catalytic domain in complex with ATP-competitive inhibitors. We determined the 1.4 Å co-crystal structure of PAK6 with the type II PAK inhibitor PF-3758309, and the 1.95 Å co-crystal structure of PAK6 with sunitinib. These findings provide new insights into the structure-function relationships of PAK6 and may facilitate development of PAK6 targeted therapies.

## Introduction

The p21-activated kinase (PAK) family comprises six sterile-20 group serine/threonine kinases. Sequence similarity and functional differences between the six members of this family has resulted in their classification as either type I (PAK1, PAK2 and PAK3) or type II (PAK4, PAK5 and PAK6) PAKs [1]. The type I PAKs are functionally and structurally well-studied, and are directly activated by interaction with Rho-family small GTPases to function in growth factor signaling and regulation of morphogenic processes [2], [3]. In contrast, the type II PAKs bind the Rho-family small GTPases CDC42, RAC1 and RhoV [3]–[5], but are not directly activated by this interaction. Instead, alternate mechanisms of activation and regulation have recently been discovered [5]–[9]. The type II PAKs are important for signaling cascades that regulate cell survival, neurite outgrowth and formation of filipodia [3].

PAK6 is expressed in prostate, testis, thyroid, placenta and neural tissues [1], [6] and is found in both cytoplasmic and nuclear fractions of prostate cells [6], [10]. Androgen receptor is reported to be a downstream target of PAK6, and PAK6 can regulate gene transcription by androgen receptor via a GTPase-independent mechanism possibly related to control of its degradation by the MDM2 E3 ubiquitin ligase [6], [11]. Global deletion of *Pak6* in mice results in increased weight and decreased aggression, possibly explained by its role in androgen receptor signaling [12]. In addition, mice with combined deletion of *Pak5* and *Pak6* show deficits in locomotion, learning and memory not associated with single deletions of either gene, suggesting functional redundancy between the two PAKs [12], [13]. While neuronal substrates specific to PAK6 have not been identified, PACSIN1 (Syndapin 1), an F-BAR protein involved in synaptic vesicle recycling, is phosphorylated redundantly by PAK4, PAK5 and PAK6 *in vivo* [14] PAK6 is overexpressed in prostate cancer [15], and its targeted inhibition could potentially decrease growth of prostate tumors [11] or sensitize prostate cancer cells to radiotherapy [16]. *PAK6* has also been found to acquire somatic mutations in other solid tumors, including mutation of residue Pro52 to leucine in two independent melanomas [17], [18]. Consequently there is increasing interest in obtaining an improved understanding the various roles of PAK6 in the cell, its substrates and autoregulation, its importance in disease and its potential targeted inhibition.

Regulation of type II PAKs was poorly understood until recently [5], [9]. Unlike many protein kinases where phosphorylation at conserved sites within the so-called ‘activation loop’ is a critical step towards full activity [19], the type II PAKs are constitutively phosphorylated in the cell [5] and not directly regulated by interaction with small GTPases, which are instead important for type II PAK relocalization [2], [20]. We, and others, identified an autoinhibitory sequence within the N-terminal region of PAK4 [5], [9] and showed by structural and biochemical analysis that this region contains a pseudosubstrate sequence centered around residue P52 [9]. Based on this work, we hypothesized that this highly conserved N-terminal region could autoinhibit each of the type II PAKs.

ATP-competitive small molecule inhibitors of the type II PAKs could be useful as cancer therapeutics [21]. The small molecule PF-3758309 was designed as a PAK4-specific inhibitor, but displays *in vitro* activity against each of the type II PAKs and also PAK1 [22]. Though effective in mouse models of cancer, it failed in human clinical trials [22]. Sunitinib (SU11248) is a potent ATP-competitive multi-kinase inhibitor that is indicated for treatment of renal cell carcinoma, imatinib-resistant gastrointestinal stromal tumors, advanced pancreatic neuroendocrine tumors and other tumor types [23], [24]. A crystal structure is available for PAK4 with PF-3758309 but none are available for a PAK family member in complex with sunitinib.

In the current study we ask whether downstream substrate specificity is conserved among the type II PAKs, whether a cancer-associated mutation that occurs in the type II PAK autoinhibitory region can activate PAK6, and whether co-crystallography might aid drug discovery for type II PAKs. By peptide array profiling we show that PAK6 has a similar substrate specificity to that previously observed for PAK4 and PAK5, implying that PAK6 may have additional substrates that overlap with other type II PAKs. We show that PAK6 kinase activity is regulated by its N-terminal pseudosubstrate *in vitro* and that a melanoma-associated mutation, P52L, in the pseudosubstrate sequence displays reduced inhibition. We go on to determine crystal structures of PAK6 kinase domain in complex with two ATP-competitive small molecule inhibitors, PF-3758309 and sunitinib. This study therefore provides molecular level details that may aid in the development of isotype specific inhibitors for the type II PAKs.

## Materials and Methods

### Cloning, Expression and Purification

Catalytic domain residues 383–674 of PAK6 (UniProt Q9NQU5) were amplified from cDNA (Open Biosystems) then subcloned into a modified pET-32 vector with an N-terminal 6xHis tag cleavable by TEV protease. A single colony of transformed *E. coli* BL21(DE3) was grown in Luria-Bertani (LB) medium at 37°C for 7 hours with kanamycin sulfate (100 µg/ml). Then the fresh culture was inoculated (1∶100) into in LB supplemented with kanamycin sulfate (100 µg/ml). Protein expression was induced at an *A*_600_ of 0.4 by addition of 0.3 mM isopropyl-thio-galactopyranoside (IPTG) at 18°C for 14 hr. Cells were harvested and then resuspended in lysis buffer (50 mM HEPES pH 7.5, 500 mM NaCl, 0.5 mM TCEP, and 1 mM PMSF). Lysozyme and DNAse-I were added and the mixture was incubated on ice before three cycles of flash-freezing in a dry ice and ethanol mixture and thawing at 4°C. Following sonication and centrifugation, the supernatant was collected and loaded onto a nickel sepharose column equilibrated with 100 ml binding buffer (50 mM HEPES pH 7.5, 500 mM NaCl, 5 mM imidazole, 0.5 mM TCEP, 5% glycerol). Contaminating proteins were washed away with 200 ml wash buffer (50 mM HEPES pH 7.5, 500 mM NaCl, 30 mM imidazole, 0.5 mM TCEP, 5% glycerol). PAK6 catalytic domain was eluted with an imidazole step gradient and identified by SDS-PAGE. Overnight TEV cleavage was performed by adding TEV protease and dialyzing the mixture against size exclusion buffer (50 mM Tris pH 8, 250 mM NaCl, 5 mM TCEP). PAK6 catalytic domain was then reloaded onto the nickel sepharose column to remove remaining uncleaved protein. The sample then was loaded onto a Superdex 75 16/60 gel-filtration column equilibrated with size exclusion buffer.

### Kinase Substrate Specificity

Peptide substrate specificity of PAK6 was determined by peptide library screening as previously described [25]. A library of 201 peptide mixtures having the general sequence Y-A-X-X-X-X-X-S/T-X-X-X-X-A-G-K-K(biotin) was used in which X represents an equimolar mixture of the 17 proteogenic amino acids excluding Ser, Thr and Cys, and S/T indicates an equimolar mixture of Ser and Thr. Each component in the library had a single amino acid residue (one of the 20 unmodified amino acids, phosphoThr or phosphoTyr) fixed at one of the nine X positions. In addition, three additional peptide mixtures, Y-A-X-X-X-X-X-S-X-X-X-X-A-G-K-K(biotin), Y-A-X-X-X-X-X-T-X-X-X-X-A-G-K-K(biotin), and Y-A-X-X-X-X-X-Y-X-X-X-X-A-G-K-K(biotin), were included to assess phosphoacceptor residue preference. Peptides (2 µl at 50 µM) were arrayed in 1536 well plates in kinase reaction buffer (50 mM HEPES, pH 7.4, 12.5 mM NaCl, 10 mM MgCl_2_, 1 mM MnCl_2_, 0.1% Tween 20). Reactions were initiated by adding PAK6 (to a concentration of 20 µg/ml) and ATP (to 50 µM with 40 µCi/ml [γ-^33^P]ATP). Plates were sealed and incubated at 30 °C for 2 hr. Aliquots (200 nl) of each reaction were then spotted onto SAM2 streptavidin membranes (Promega), which were washed as described and air-dried. Radiolabel incorporation into each peptide was determined by phosphor imaging and quantified using QuantityOne software (Bio-Rad).

### *In vitro* PAK6 Kinase Assay

Kinase activity was assessed using a radioactive assay with myelin basic protein (MBP) as substrate. The activity of recombinant PAK6 catalytic domain (residues 383–674) purified as described above, was considered optimally active, and the effects of synthesized peptides were tested. Kinase assays were performed by adding 150 nM of PAK6, 2 µM of MBP as the substrate, 50 µM of cold-ATP and 0.1 µCi of hot [γ-^33^P] ATP in Tris buffer (20 mM Tris-HCl pH 8.0, 0.15 M NaCl, 1 mM DTT and 10 mM MgCl_2_) for a total volume of 25 µl. Trans-inhibition assays performed using between 0.06 mM and 5.85 mM of purified wild-type (RRPKPVVDP) or P52L (RRPKLVVDP) peptides. Control peptide (APRTPGGRR) was purchased from Anaspec and inhibition assays were performed using between 0.5 mM and 4.5 mM of peptide. The reaction was conducted at 30°C for 10 min and stopped by addition of 5X sample buffer and analyzed by SDS-PAGE. Gels were analyzed by exposure to phosphor storage screen (GE) followed by scanning using a Molecular Imager FX Pro Plus System (Bio-Rad) and quantification by optical densitometry Quantity One (Bio-Rad). Relative activities were compared to those measured for PAK6. Measurements were calculated from at least three independent experiments.

### Cell-based Assay

HEK293 cells were grown at 37°C with 5% CO_2_ in Dulbecco’s modified Eagle’s medium (DMEM) supplemented with 10% fetal bovine serum and penicillin/streptomycin. Cells were transfected with pCMV6-myc-PACSIN1 (kindly provided by J. Peterson) and pEGFP-C2, pEGFP-PAK6-WT, pEGFP-PAK6-P52L, pEGFP-PAK6-N (N-terminus, residues 1–382) or pEGFP-PAK6-CAT (catalytic domain, residues 383–674) using polyethylenimine (PEI, Polysciences Inc.). Twenty four hours later, cells were trypsinized, quenched with complete medium, washed with PBS and lysed in buffer containing 20 mM Tris (pH 8.0), 200 mM NaCl, 1 mM EDTA, 1% Triton X-100, Complete Protease Inhibitor Cocktail (Roche), and phosphatase inhibitors (1 mM Na_3_VO_4_, 10 mM NaF, 10 mM β-glycerol phosphate) for 10 minutes at 4°C. Samples were resolved by SDS-PAGE and were assessed by immunoblotting using the following antibodies: goat anti-GFP (1∶1000; Rockland), mouse anti-myc (9E10, 1∶1000; Santa Cruz Biotechnology), rabbit anti-phospho-PACSIN1 (1∶2000; gift of J. Peterson), mouse anti-vinculin (1∶10,000; Sigma), anti-goat or anti-rabbit IgG AlexaFluor680 (1∶5000, Invitrogen), anti-mouse IgG IRDye800 (1∶5000, Li-Cor). Bands visualized using a Li-Cor Oddysey imager and were quantified using ImageJ. A ratio of phospho-PACSIN1 to total PACSIN1 was calculated by dividing the total phospho-PACSIN1 signal by the myc signal.

### Crystallization and Data Collection

PAK6 kinase domain was concentrated to between 8 and 13 mg/ml and incubated on ice overnight at a 1∶3 molar ratio with PF-3758309 or sunitinib (gifts from Joseph Schlessinger) (dissolved in DMSO). Initial crystallization screens were conducted using sparse matrix crystallization kits (Qiagen) and crystallization trays were set with a Matrix Hydra-II eDrop crystallization robot (Thermo Scientific). Following detailed grid screening to optimize conditions for both PAK6:inhibitor co-crystals were obtained overnight at 4°C using sitting drop vapor diffusion methodology. Co-crystals of PAK6 in complex with PF-3758309 grew in 30% (*v*/*v*) isopropanol, 0.1 M bicine pH 9.0 in sitting drop morphology. Co-crystals of either inhibitor with PAK6 were obtained using 30% (*v*/*v*) isopropanol, 0.1 M MES (pH 6.0). For cryoprotection, crystals were briefly soaked in the precipitant well conditions supplemented with 30% MPD before flash freezing. Crystallographic data were collected at beamline ID24-E at the Advanced Photon Source for the co-crystal of PAK6 with PF-3758309, and at beamline X6A at the National Synchrotron Light Source for the co-crystal of PAK6 with sunitinib.

### Structure Solution and Refinement

A 1.40 Å dataset was collected for the co-crystal of PF-3758309 with PAK6 and a 1.95 Å dataset for the co-crystal of PAK6 with sunitinib. The structures were determined using software installed and configures by SBGrid [26]. Diffraction data were processed using HKL2000 [27]. Each structure was determined by molecular replacement using Phaser [28], with the previously determined catalytic domain of PAK6 (PDB ID: 2C30) [29] used as the search model structure. Automatic model building was performed using ARP/wARP to mitigate model bias [30]. Following several rounds of refinement and manual rebuilding using REFMAC5 [31] or Phenix [32] and Coot [33] clear difference density allowed easy placement of the inhibitors PF-3758309 or sunitinib into the respective structures. Final converged models of the co-crystal structures were then obtained following several further rounds of manual rebuilding and refinement using Coot, REFMAC5, or Phenix with TLS. The final models were validated using MolProbity [34]. Both crystal structures have good electron density and both inhibitors are clearly defined. Data collection and refinement statistics are presented in **Table 1**.

**Table 1:** Data collection, phasing, and refinement statistics.

	PAK6 with PF-3758309	PAK6 with sunitinib
**PDB ID**	4KS7	4KS8
**Data Collection**		
Number of crystals	1	1
Space Group	*P*2_1_2_1_2_1_	*P*2_1_2_1_2_1_
X-ray source	APS ID24-E	NSLS X6A
Cell dimensions, *a, b, c* (Å)	48.6, 59.7, 100.5	49.6, 60.1, 100.0
* α, β, γ* (°)	90, 90, 90	90, 90, 90
Wavelength (Å)	0.9792	1.0000
Resolution range (Å)^a^	50–1.4 (1.45–1.40)	50–1.95 (2.02–1.95)
No. unique reflections	57856	22562
Degrees of data (°)	180	180
Completeness (%)^a^	99.8 (99.6)	100.0 (100.0)
*R*_sym_ (%)^a^	7.1 (82.5)	11.7 (88.5)
*<I>/*<σ(*I*)>^a^	20.0 (1.6)	17.5 (2.9)
Redundancy^a^	7.0 (6.1)	6.8 (6.7)
Wilson *B*-factor (Å^2^)	16.1	20.0
**Refinement**		
Resolution Range (Å)^a^	50–1.4 (1.44–1.40)	38.5–1.95 (2.00–1.95)
*R* factor (%)^a^		
Working set	19.3 (31.5)	21.8 (30.4)
Test set	21.7 (34.3)	26.1 (27.2)
Residue range built	386–672	385–671
No. non-hydrogen atoms (total)	2685	2496
Protein	2388	2296
Water	258	171
Inhibitor	35	29
Other solvent (isopropanol)	4	–
Overall *B*-factor (Å^2^)	22.0	28.1
Protein	21.0	27.8
Water	31.0	28.8
Inhibitor	19.6	40.8
Other solvent (isopropanol)	30.6	–
**Model Quality**		
RMSD bond lengths (Å)	0.016	0.006
RMSD bond angles (°)	2.034	1.337
MolProbity		
Score	1.62	1.13
Percentile	72^nd^	100^th^
Ramachandran plot (%)	98.0/2.0/0	97.5/2.5/0
favored/allowed/outliers		

a Parentheses indicate highest resolution shell.

## Results

### PAK6 Substrate Specificity Profiling

**Figure 1:**
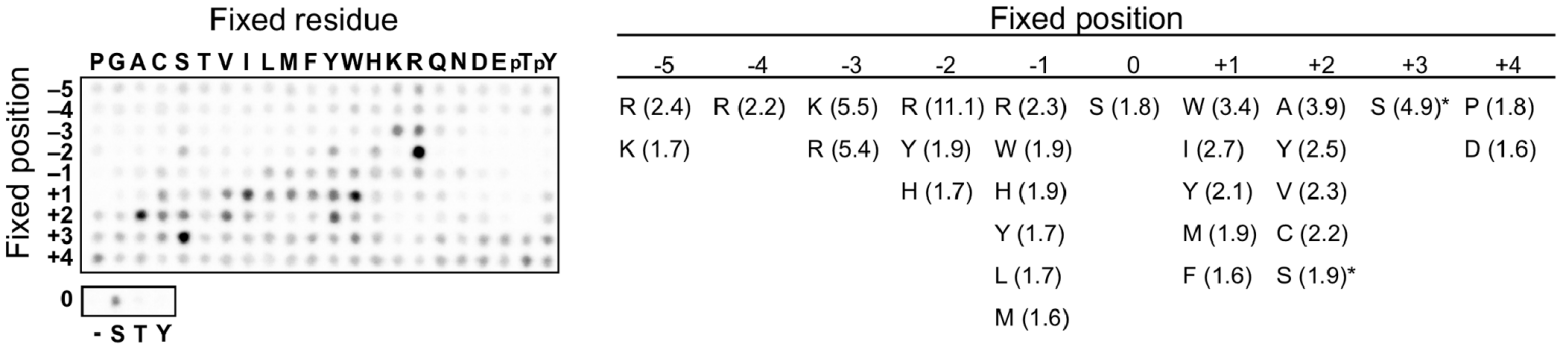
Peptide library analysis of PAK6 phosphorylation site specificity. Left panel, relative phosphorylation levels of 201 peptide mixtures within a positional scanning peptide library by PAK6. PAK6 was incubated with the peptide mixtures in the presence of [γ-^33^P]ATP, and radiolabel incorporation was assessed by phosphor imaging. A representative image of two replicates is shown. Right panel, positively selected amino acid residues at each position. Quantified spot intensities from two separate runs were background corrected and then normalized so that the average value within a position was 1.0. Residues with normalized selectivity values greater than 1.6 are shown. Values are the mean of two runs. An asterisk indicates strong apparent selection for Ser that may be an artifact due to peptide mixtures with fixed Ser residues having two potential sites of phosphorylation.

Unique functions of PAK6 are likely to be mediated in part by specific downstream substrates. We therefore wondered whether differences in substrate specificity could be predicted for PAK6 when compared to known targets of the other type II PAKs, PAK4 and PAK5 (PAK7). The phosphorylation site specificity of PAK1 and PAK2 (type I PAKs) as well as PAK4 and PAK5 (type II PAKs) has been investigated using individual short peptide substrates and peptide libraries, but the substrate specificity of PAK6 has not been investigated [14], [35], [36]. We utilized a positional scanning peptide library approach to compare substrate specificity at the peptide level for PAK6 with the other PAKs. PAK6 was incubated with an array of 201 peptide substrate mixtures. Each peptide in the array contains a fixed central serine or threonine residue as the phosphoacceptor and a fixed amino acid at one of the nine surrounding positions [37]. Analysis of the full set of peptides in parallel allows us to quantitatively examine the effect of substituting each of the 20 amino acids at each position within the peptide sequence on PAK6 activity. Previously, both type I and type II PAKs were found to display a distinct preference for basic residues two and three amino acids N-terminal to the phosphoacceptor and a preference for hydrophobic residues immediately downstream of the phosphorylation site. Our peptide array analysis shows similar preferences for PAK6 (**Fig 1** **;** quantified data are presented in **Table S1**). As with the other PAKs, PAK6 also had a strong preference for serine over threonine as the phosphoacceptor residue, with no detectable phosphorylation of a peptide containing only tyrosine at the phosphorylation site [14], [36]. A previous study also noted that type II PAKs display preferences for Ala and Ser respectively two and three residues downstream of the phosphorylation site. As anticipated, PAK6 was found to have these additional preferences as well. Overall, these similarities in phosphorylation site preference among PAK family members suggest that differences in substrate specificity for the family members are likely due more to differences in spatial-temporal localization and expression of the kinases than to distinct preferences for substrate governed by the kinase domains themselves. Furthermore, the similarities in substrate specificity may suggest potential overlap in downstream substrates for the type II PAK family members.

### Inhibition of PAK6 by its N-terminal Pseudosubstrate

**Figure 2:**
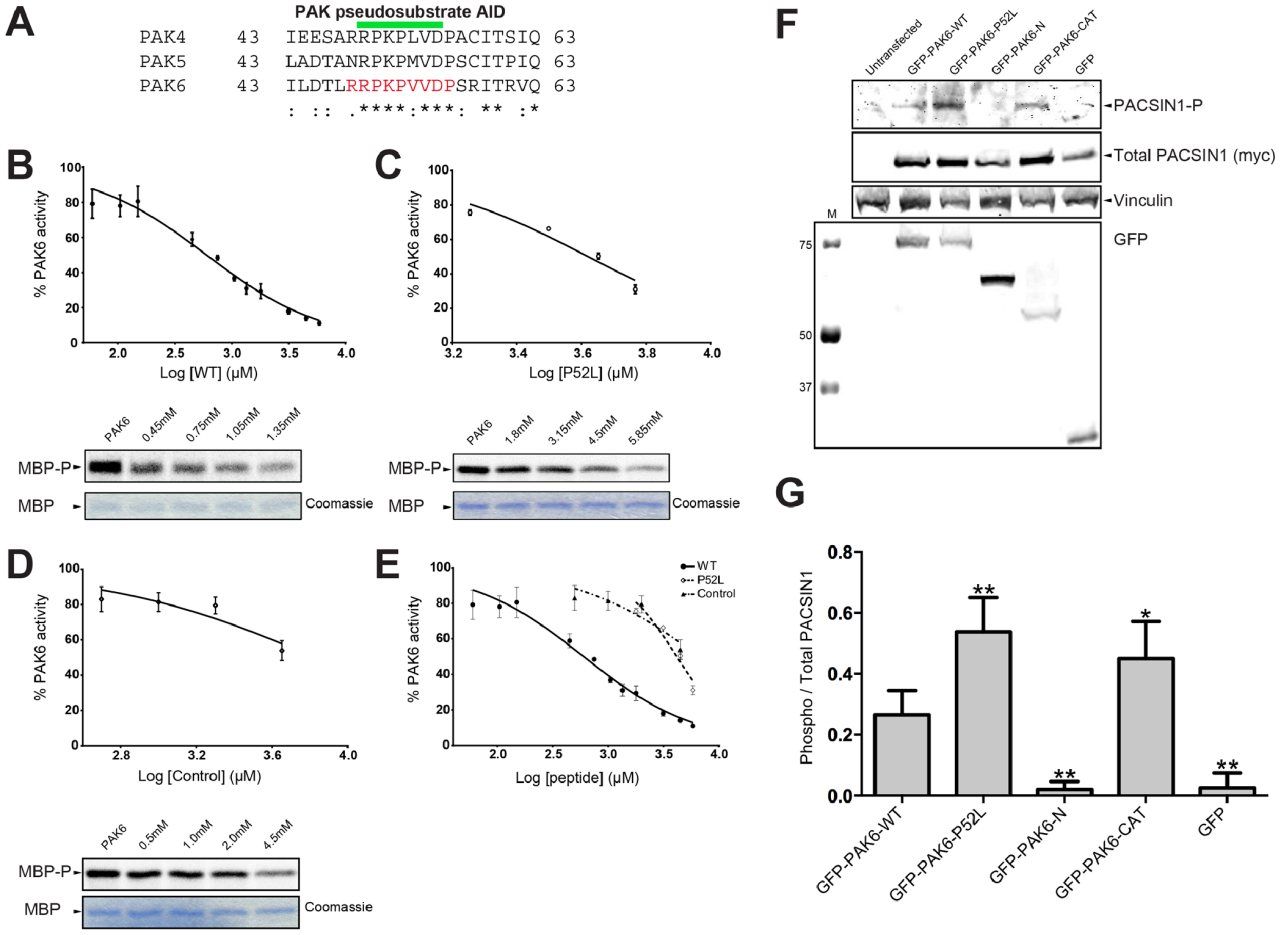
P52L mutation results in loss of PAK6 autoinhibition. **A)** Alignment of the pseudosubstrate autoinhibitory domain (AID) of the type II PAK family members is shown. Pseudosubstrate region indicated by a green bar. Peptide used in panel B indicated in red. Conservation shown with “*” indicating identical, “:” indicating highly conserved and “.” Indicating semi-conserved. **B–D)** Dose-dependent relationship of the inhibition of wild-type (RRPKPVVDP), P52L (RRPKLVVDP) mutant, and control (APRTPGGRR) peptides on PAK6 kinase activity. **B)** Inhibition of PAK6 by wild-type (WT) peptide shown on a log scale. Data shows a mean and S.E.M error bar with 3 independent experiments. Concentration range from 0.06 mM to 5.85 mM of peptides was tested. **C)** Inhibition of PAK6 by P52L peptide shown on a log scale. Data shows a mean and S.E.M error bar with 3 independent experiments. Concentration range from 1.8 mM to 5.85 mM of peptides was tested. **D)** Inhibition of PAK6 by a control peptide. Data show a mean and S.E.M error bar with 3 independent experiments. Concentration range from 0.5 mM to 4.5 mM of peptide was tested. **E)** Inhibition plots from panels B-D shown on the same graph. **F)** Co-transfection of PAK6 with PACSIN1. Co-transfection of myc-PACSIN1 and GFP-PAK6 constructs probed for PACSIN1 phosphorylation using a phospho-specific PACSIN1 antibody. M indicates molecular weight marker. **G)** Quantification of E. Ratio of phosphorylated PACSIN1 to total PACSIN1 expressed is shown, normalized for Phospho PACSIN1/Total PACSIN1 (myc). PACSIN1 shows significantly increased phosphorylation when co-transfected with the P52L mutant compared to the wild-type PAK6 (* indicates p<0.05; ** indicates p<0.01; Student’s t-test). n = 4. Error bars indicate S.E.M.

We next wondered whether PAK6 autoinhibition occurs in a similar manner as previously observed for PAK4 [9]. The pseudosubstrate autoinhibitory domains (AID) of the type II PAKs are well conserved (**Fig 2A**) and we previously showed that PAK6 can be inhibited by the PAK4 autoinhibitory pseudosubstrate [9]. We therefore tested whether the presumed N-terminal pseudosubstrate of PAK6 would inhibit kinase activity of the PAK6 catalytic domain. We conducted kinase assays using myelin basic protein (MBP) as a substrate, constant amounts of PAK6 catalytic domain and increasing concentrations of a peptide corresponding to the PAK6 N-terminal pseudosubstrate (R^48^RPKPVVDP). Following incubation for 10 minutes with radiolabeled ATP we performed SDS-PAGE on the reaction mixtures and analyzed the incorporation of radioactivity into MBP by autoradiography. We found that PAK6 was inhibited by its N-terminal peptide with an IC_50_ of 600 µM (95% confidence: 510–710 µM)(**Fig 2B**).

### Loss of PAK6 Inhibition by a Cancer-associated Mutation

Somatic acquired PAK6 point mutations have been discovered in cancer, and interestingly, a recurrent PAK6 mutation that has been observed in two independent melanomas [17], [18] occurs by substituting residue P52 within the pseudosubstrate autoinhibitory region with a leucine. We previously postulated that this recurrent mutation may interrupt PAK6 pseudosubstrate autoinhibition [9]. To test whether this might be the case we conducted kinase activity assays as before, but with increasing concentrations of the P52L mutant peptide (R^48^RPKLVVDP). This showed that the P52L mutant pseudosubstrate peptide was severely impaired in its ability to inhibit PAK6 (**Fig 2C**), with an IC_50_ of 4200 µM (95% confidence: 3800–4600 µM), a 7-fold weaker inhibition than observed with the wild-type pseudosubstrate peptide. A control peptide (APRTPGGRR), displays a similar IC_50_ (6700 µM; 95% confidence: 3000–15000 µM) to that observed for the P52L mutant (**Fig 2D and E**). This suggests that the P52L mutant of PAK6 may result in significant reduction of PAK6 autoinhibition in these melanoma tumors, and residual inhibition by the P52L substituted peptide in vitro may be attributable to weak, non-specific interactions.

To investigate whether the P52L mutation impaired full-length PAK6 auto-inhibition in cells, we examined PAK6 phosphorylation of PACSIN1, an established type II PAK substrate [14]. Co-transfection of myc-PACSIN1 with full-length WT GFP-PAK6 in HEK293 cells led to increased phosphorylation of PACSIN1 at Ser343 compared to co-transfection with either GFP alone or GFP fused to the N-terminal non-catalytic region of PAK6 as assessed with a, phospho-specific PACSIN1 antibody [14]. We find that PACSIN1 shows significantly increased phosphorylation when co-transfected with the P52L mutant compared to the wild-type PAK6 (**Fig 2F and G**), suggesting that the melanoma-associated mutation indeed functions to increase kinase activity.

### Overall Analysis of the Crystal Structures of PAK6 in Complex with PF-3758309 and Sunitinib

**Figure 3:**
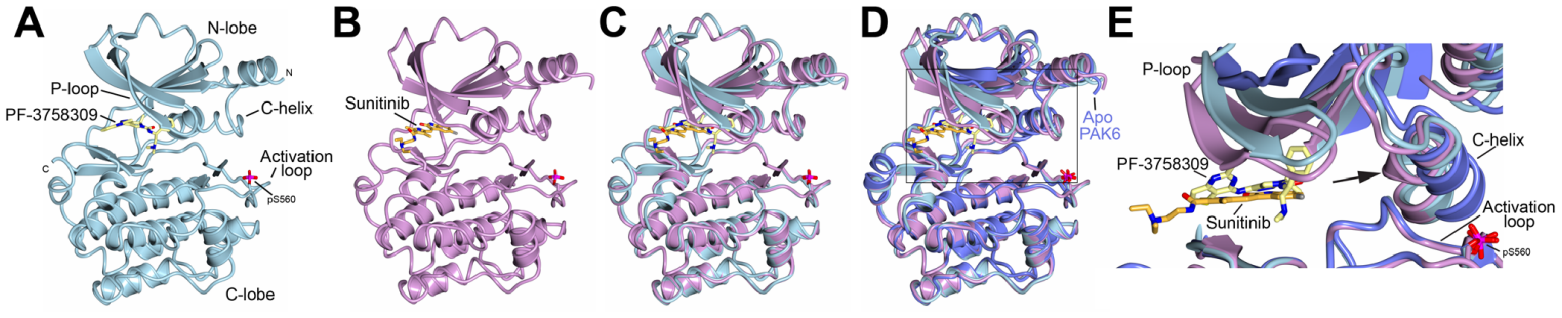
Crystal structures of PAK6 with PF-3758309 and with sunitinib. **A)** Overall structure of PAK6 in complex with PF-3758309. PAK6 shown in cartoon format in cyan, PF-3758309 shown in stick format with carbons shown yellow. PAK6 N-lobe, C-lobe, activation loop, C-helix and P-loop are labeled. N- and C- termini are indicated. Phosphorylated activation loop serine, pS560, is shown in stick format and labeled. **B)** Overall structure of PAK6 in complex with sunitinib. PAK6 is colored purple and carbon atoms of sunitinib are colored orange. Global structural features are as indicated in **A**. **C)** Superposition of PAK6 with PF-3758309 and PAK6 with sunitinib. Slight conformational differences are observed in the glycine-rich P-loop, the C-helix and the orientation of the N-lobe. Superposition is of the kinase domain the C-lobes. **D)** Superposition of PAK6 with PF-3758309, PAK6 with sunitinib and apo PAK6. Crystal structure of Apo PAK6 [29] (PDB ID: 2C30) superposed onto the two inhibitor-bound PAK6 structures. Significant conformational movements are observed in the N-lobe particularly around the glycine-rich P-loop and the C-helix. Boxed area indicates the zoomed region shown in **E**. **E)** Close up highlighting the conformational differences between the PAK6 structures. A kink in the C-helix is clearly visible for the PF-3758309 and sunitinib-bound structures, indicated with an arrow. All structural figures generated using CCP4mg [46].

We then conducted a crystallographic study to investigate the interactions of PAK6 with two ATP-competitive inhibitors, PF-3758309 and sunitinib. We obtained crystals of PAK6 kinase domain in complex with either PF-3758309 or sunitinib by co-crystallization. These co-crystals diffracted X-rays to Bragg spacings of 1.40 Å and 1.95 Å respectively for the PF-3758309 and sunitinib co-crystals. The overall crystal structures displayed the canonical bi-lobed protein kinase fold, with a predominantly β-strand N-lobe and a predominantly α-helical C-lobe (**Fig 3A and 3B**). The ATP-binding site is located between the lobes and is occupied by the small molecule inhibitors. Good electron density is observed throughout the structures and the conformations of the small molecules are clearly observed. Both co-crystals were obtained in the same crystal form with the activation loop in the extended conformation and residue S560 phosphorylated. Kinases are often classified by the conformation of three residues at the amino-terminus of the activation loop, Asp-Phe-Gly [38], [39], and we observe that the DFG flip status for both PAK6 crystal structures is ‘DFG-in’. When the N- and C-lobes are superposed the root-mean-square deviation (RMSD) for the N-lobes is 0.48 Å over 98 Cα atoms and for the C-lobes is 0.39 Å over 187 Cα atoms. However, the overall RMSD is 0.82 Å over 285 Cα atoms, illustrating a slight conformational rotation in the kinase N-lobe between the structures (RMSDs calculated using TOPP [40])(**Fig 3C**).

### Comparison with Previous apo PAK6 Structure

A crystal structure of the kinase domain of PAK6 has previously been determined in the apo form with an open conformation [29] (PDB ID: 2C30). Comparison of the apo PAK6 structure with PAK6:PF-3758309 and PAK6:sunitinib yields RMSDs of 1.10 Å over 244 Cα atoms and 1.14 Å over 236 Cα atoms, respectively (**Fig 3D**). Notably, the C-lobes are very similar between the apo and inhibitor-bound PAK6 structures with RMSD of 0.44 Å over 188 Cα atoms between the apo and PAK6:PF-3758309 structures and 0.53 Å over 187 Cα atoms between the apo and PAK6:sunitinib structures. In contrast, the N-lobe of the apo PAK6 shows higher deviation from the inhibitor-bound structures, with RMSD of 1.28 Å over 86 Cα atoms between the apo and PAK6:PF-3758309 structures and 1.38 Å over 84 Cα atoms between the apo and PAK6:sunitinib structures. The conformational flexibility accessible by PAK6 is therefore not solely due to opening and closing of the N-lobe, but by conformational differences within this lobe. These are centered on two regions, the glycine-rich P-loop and the C-helix (**Fig 3E**). For the two inhibitor-bound PAK6 structures the P-loop is closed, which is divergent from the apo PAK6 structure. The inhibitor-bound PAK6 structures also exhibit a shift in the C-helix, similar to that previously observed for type II PAKs [29]. Furthermore, for both structures the N-terminus of the C-helix (residues 446 to 450 in the sunitinib-bound structure and residues 446 to 448 in the PF-3758309-bound structure) fold as a 3_10_ helix and the catalytic glutamic acid, E452, is extended to hydrogen bond to K436.

### PAK6 in Complex with PF-3758309

**Figure 4:**
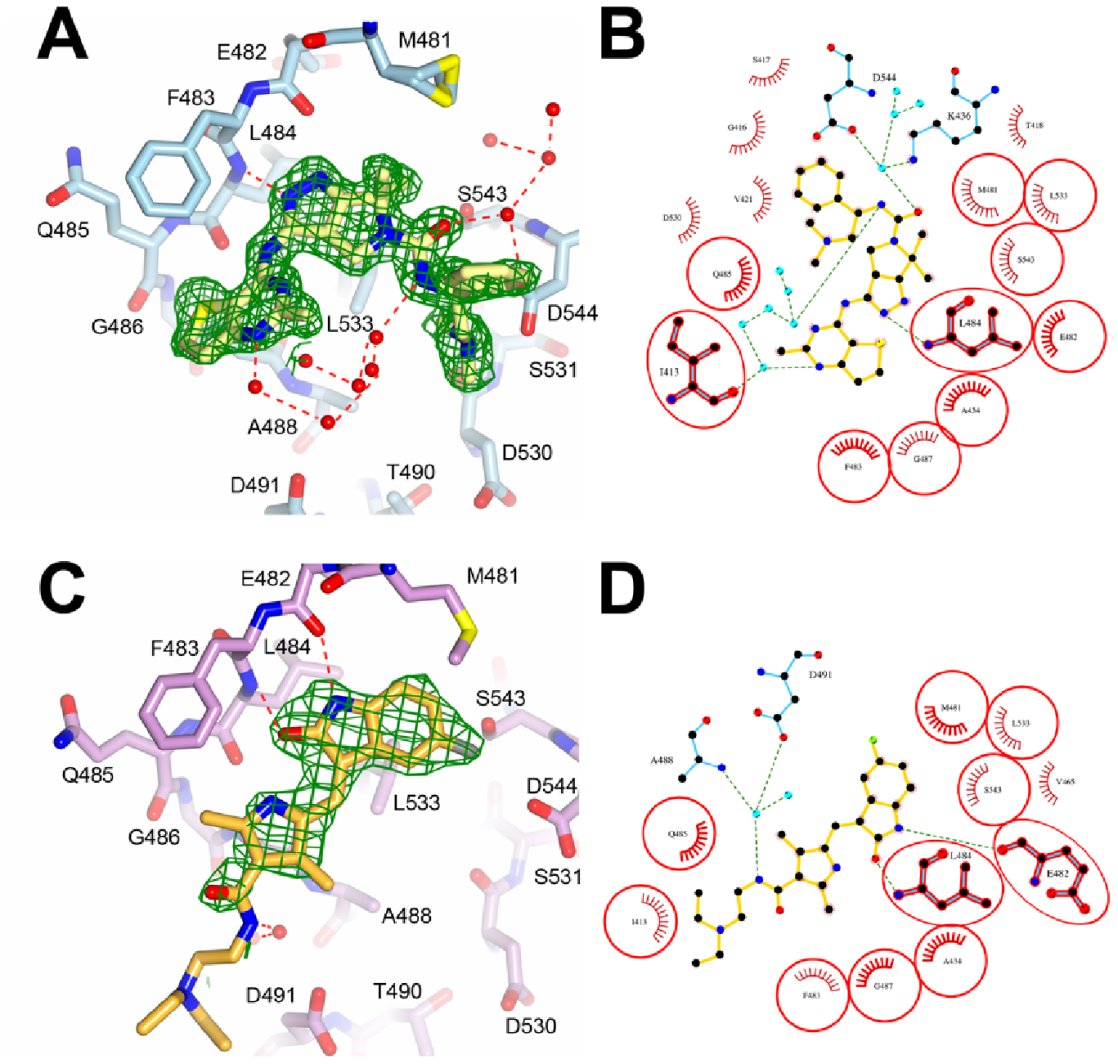
Interations of PF-3758309 and sunitinib with PAK6. **A)** Overview showing the interactions of PF-3758309 with PAK6. Residues are shown in stick format and labeled. PF-3758309 shown in stick format with carbons shown yellow. Hydrogen-bonds shown as red dashed lines. Unbiased *F*_obs_-*F*_calc_ electron density is shown for PF-37508309 in green at 3 σ. Electron density is from the final round of refinement prior to building PF-3758309. The mode of binding is clearly visible and compatible with the final refined orientation of PF-3758309. For clarity the map is clipped at 3 Å from PF-3758309. M481 is observed in two conformations, which are both shown. Waters indicated by red spheres. **B)** Ligplot+ [47] schematic showing the interactions of PAK6 and PF-3758309. PAK6 residues that make contact with both PF-3758309 and sunitinib are highlighted in red. Waters indicated by cyan spheres. **C)** Overview showing the interactions of sunitinib with PAK6. Residues are shown in stick format and labeled. Sunitinib shown in stick format with carbons shown orange. Hydrogen-bonds shown as red dashed lines. Unbiased *F*_obs_-*F*_calc_ electron density shown for sunitinib in green at 3 σ. Electron density is from the final round of refinement prior to building sunitinib. The mode of binding is clearly visible and compatible with the final refined orientation of sunitinib. For clarity the map is clipped at 3 Å from sunitinib. Waters indicated by red spheres. **D)** Ligplot+ [47] schematic showing the interactions of PAK6 and sunitinib. PAK6 residues that make contact with both PF-3758309 and sunitinib are highlighted in red. Waters indicated by cyan spheres.

The 1.4 Å co-crystal structure of PF-3758309 in complex with PAK6 highlights the mechanism of binding for this small molecule to a second PAK family member (**Fig 4A**). Previously a co-crystal structure of PF-3758309 was determined to 2.1 Å resolution in complex with PAK4 [22]. Overall, the PAK6 co-crystal structure displays a slightly more open conformation of the N-lobes when compared to the PAK4 co-crystal structure, however, the mode of small molecule binding between PAK6 and PAK4 is found to be very similar. The PAK4 co-crystal structure had shown an ATP-competitive mode of binding that is mediated by multiple hydrogen-bonds between the kinase linker region and the pyrrolopyrazole core, amine linker and thienopyrimidine ring; each of these hydrogen-bonds is conserved in the PAK6 co-crystal structure. Further hydrogen bonds were also observed from the urea carbonyl via a conserved water molecule to PAK4 residues K350 and D458, and from the dimethylamine group to PAK4 residue D458. These hydrogen bonds are also conserved in the PAK6/PF-3758309 co-crystal structure and are mediated by K436, D544 and a water molecule in a very similar location. The hydrophobic interactions noted between PF-3758309 and PAK4 are recapitulated in the PAK6 co-crystal structure, these include between the pyrimidine C2 methyl group and PAK4 residues G328 and V335 (PAK6 residues G414 and V421), as well as the thiophene and G401 (PAK6 residue G487) (**Fig 4B**). Interestingly, however, we do observe conformational variability in the interactions between the gem-dimethyl group of the pyrrolopyrazole core and the PAK gatekeeper methionine. In contrast to the PAK4 co-crystal structure, the gatekeeper residue M481 in the PAK6 co-crystal structure is clearly observed in two alternate conformations, potentially indicating some conformational flexibility in this region, although it is not clear whether this would represent a usable difference to aid selectivity design for PAK-specific small molecules.

### PAK6 in Complex with Sunitinib

Sunitinib displays selectivity for type II over type I PAK family members, but is a weak inhibitor of the PAK kinases overall (*K*_i_ is estimated in the range of 500–7000 nM for type II PAKs) [41]. Therefore understanding how sunitinib binds to the type II PAKs may represent a starting point for future small molecule drug discovery. There are currently 6 co-crystal structures of protein kinases in complex with sunitinib deposited in the PDB. These kinases are of VEGFR (4AGD) [41], KIT (3G0E) [42], KIT-D816H mutation (3G0F) [42], ITK (3MIY) [43], CDK2 (3TI1) [44] and phosphorylase kinase γ2 (2Y7J) (unpublished). In each of these structures the dihydrooxaindole and pyrrole rings of sunitinib make hydrogen-bonds to the kinase linker region, a pattern that is replicated in the PAK6 co-crystal structure. van der Waals interactions are made between the 5-fluoro-2-oxo-1H-indol-3-ylidene and pyrrole rings and M481 (the gatekeeper residue), V465, L533, A434, V421, I413 and G487. These interactions allow the sunitinib ring system to slot neatly into the adenosine-binding cleft (**Fig 4C**). In each of the sunitinib-bound structures the aliphatic diethylaminoethyl tail of sunitinib is exposed to solvent. This is also the case for the PAK6 co-crystal structure where the aliphatic tail of sunitinib is visible in the electron density and can be built (**Fig 4D**).

### Conservation Analysis of Type II PAKs

**Figure 5:**
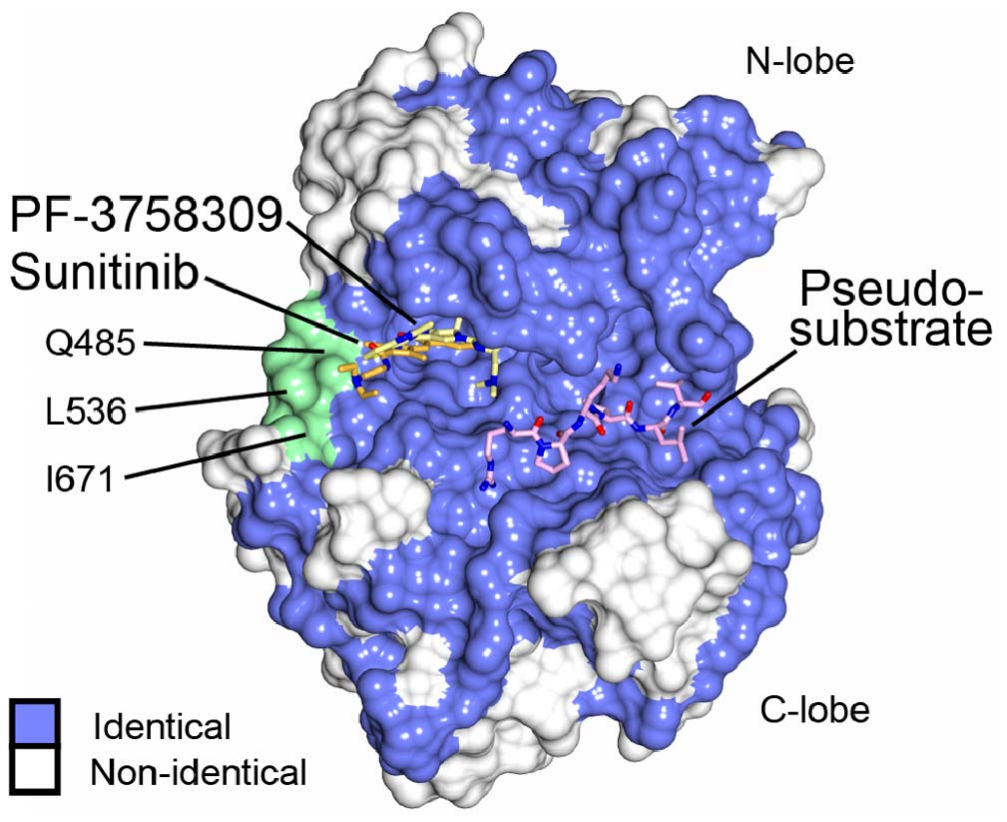
Surface conservation of the type II PAKs. PAK6 in complex with PF-3758309 is shown with the surface of PAK6 protein colored blue for residues identical between human PAK4, PAK5 and PAK6, and white for non-conserved residues. PF-3758309 is shown in stick format with carbons shown yellow. The co-crystal structures of PAK6 with sunitinib and PAK4 in complex with its autoinhibitory pseudosubstrate [9] (PDB ID: 4FII) were superposed onto the PAK6/PF-3758309 structure and both sunitinib and the autoinhibitory pseudosubstrate inhibitor are shown. The carbon atoms of sunitinib are colored orange and the carbon atoms of the PAK4 autoinhibitory pseudosubstrate are colored pink. Residues adjacent to the kinase linker display differences between the type II PAK family, and are labeled and colored green. Kinase N- and C-lobes are indicated.

In view of the similarities of substrate specificity profile, kinase autoinhibition and kinase inhibitor binding among the type II PAKs we conducted a surface conservation analysis to investigate whether there are amino differences that could indicate altered substrate specificity or unexplored locations to obtain a PAK isoform-specific small molecule inhibitor. On analysis of the conservation among the type II PAKs, the residues that determine peptide substrate specificity are completely conserved (**Fig 5**). This correlates well with our finding that the kinase domain is autoinhibited by its pseudosubstrate sequence (**Fig 2**), and that the substrate specificity is similar to PAK4 (**Fig 1**). Therefore we conclude that spatial-temporal kinase localization is a primary driver for regulation of PAK6 and for all of the type II PAK family members. For inhibitor binding we note that there is a region adjacent to the kinase linker that displays amino acid differences between the PAK family members (**Fig 5**). These residues include Q485 (E399 in PAK4, E527 in PAK5), L536 (H450 in PAK4, S578 in PAK5) and I671 (M585 in PAK4, M713 in PAK5) and are immediately adjacent to the C-terminus of the type II PAK kinase domain, which is also divergent. There is therefore the potential that small molecule specificity between the type II PAKs could be obtained by utilizing amino acid differences in this region.

## Discussion

Both androgen receptor and MDM2 have been suggested to be unique substrates of PAK6 [6], [11], [45], and PACSIN1 is a shared substrate of the type II PAKs. However, the unique phenotypes associated with *PAK6* deletion imply that additional specific PAK6 substrates remain to be discovered. The mode of regulation of PAK6 is thought to be similar to that previously identified for PAK4, but this has not been shown with a cognate PAK6 sequence. Furthermore, the impact of somatic, acquired cancer mutations in PAK6 has not been studied, and structural biology techniques to identify potential ways to specifically target dysregulated type II PAKs have not yet been successful. In this study we have addressed each of these specific questions regarding PAK6 signaling.

We have conducted an investigation into the substrate specificity of PAK6. We find that PAK6 has an identical consensus phosphorylation site sequence to PAK4 and PAK5 [14], [36]. While PAK5 and PAK6 appear to be partially redundant, they are thought to have functions distinct from PAK4. Given that these three kinases share the same phosphorylation consensus, it seems likely that targeting of specific downstream substrates involves interactions outside of the kinase active site, perhaps mediated by accessory proteins. As noted previously, the PAK5/PAK6 phosphorylation site in PACSIN1 (Ser343, with the surrounding sequence DRG**S**VSS) conforms well to the type II PAK consensus sequence, having an Arg residue at the P -2 position, a Val residue at the P +1 position, and a Ser phosphoacceptor residue. By contrast, the sequence surrounding the PAK6 phosphorylation site in androgen receptor (Ser578, TCG**S**CKV) does not conform to the consensus, suggesting a role for non-active site interactions in directing phosphorylation of androgen receptor. The general type II PAK consensus is overall similar to the type I PAK consensus sequence as determined for PAK1 and PAK2. However, phosphorylation of PACSIN1 specifically by type II PAKs can be explained by phosphorylation site recognition by the kinase catalytic domain [14]. These observations suggest that differences between type I and type II PAKs in their preferred phosphorylation site sequences are important for specific substrate targeting *in vivo*. We discovered that PAK6 is inhibited by its pseudosubstrate sequence. Our previous studies had shown that PAK6 could be inhibited by the PAK4 pseudosubstrate sequence [9], so our current study builds on our previous finding to show that the PAK6 pseudosubstrate sequence can inhibit its own kinase activity. We also find that a melanoma-associated mutation within the pseudosubstrate sequence, P52L, disrupts PAK6 autoinhibition enhancing its kinase activity, potentially correlating with increased expression of PAK6 in prostate cancer, and implied alterations in kinase activity [15]. Establishing that this mutation impacts pseudosubstrate autoinhibition may facilitate future PAK6 studies. Finally, we determined two co-crystal structures of PAK6 in complex with ATP-competitive small molecule inhibitors. These co-crystal structures will facilitate an improved understanding of the modes of targeted inhibition for type II PAKs, and may aid future studies that aim to design inhibitors specific to each of the type II PAK family members.

In sum, the current study provides significant advances in the understanding of the type II PAK family member, PAK6, and will facilitate future studies into PAK signaling and targeted drug development.

## Supporting Information

Table S1 **Quantification of peptide library assay.** Normalized quantified peptide library data. Data are normalized so that the average value with a position is 1.0. The average values from two separate runs are shown. Cells with values greater than 1.6 are shaded green.
(PDF)
