# Supplementary material for: Substrate and Inhibitor Specificity of the Type II p21-Activated Kinase, PAK6
Source: PLoS One. 2013 Oct 28;8(10):e77818. doi: 10.1371/journal.pone.0077818 (PMC3810134; doi:10.1371/journal.pone.0077818)
Supplement: Table S1 — Quantification of peptide library assay. Normalized quantified peptide library data. Data are normalized so that the average value with a position is 1.0. The average values from two separate runs are shown. Cells with values greater than 1.6 are shaded green. (PDF) [file pone.0077818.s001.pdf]

Table S1. **Quantification of peptide library assay.** Normalized quantified peptide library data. Data are normalized so that the average value with a position is 1.0. The average values from two separate runs are shown. Cells with values greater than 1.6 are shaded green.

	Position									
	-5	-4	-3	-2	-1	0	+1	+2	+3	+4
P	0.93	0.77	0.33	0.47	0.14		0.01	0.59	0.76	1.80
G	1.18	1.08	0.42	0.07	0.43		0.12	0.51	0.93	0.75
A	0.70	0.78	0.38	0.24	0.65		0.26	3.91	0.74	1.10
C	0.66	1.01	0.46	0.17	0.83		1.19	2.21	1.27	0.52
S	1.45	1.01	1.06	1.58	0.62	1.81	0.72	1.90	4.94	1.18
T	1.07	0.75	0.35	0.50	0.39	0.19	0.73	0.68	0.52	0.68
V	0.97	0.61	0.33	0.35	0.36		1.47	2.30	0.75	1.06
I	0.69	0.69	0.28	0.22	0.42		2.74	1.00	0.70	0.84
L	0.87	0.78	0.34	0.19	1.71		1.55	0.50	0.84	0.67
M	1.15	0.96	0.47	0.34	1.62		1.94	0.33	0.86	0.83
F	0.68	0.89	0.51	0.51	1.41		1.60	0.55	0.89	1.03
Y	0.65	1.07	0.41	1.85	1.73		2.09	2.49	0.86	0.90
W	0.79	1.46	0.71	0.27	1.94		3.42	0.97	1.36	1.15
H	0.94	1.19	0.72	1.68	1.85		0.70	0.48	0.74	1.18
K	1.73	1.32	5.51	0.24	1.46		0.25	0.11	0.21	0.63
R	2.37	2.17	5.43	11.05	2.28		0.52	0.15	0.37	0.57
Q	0.87	0.96	1.23	0.13	0.68		0.41	0.30	0.80	1.22
N	0.84	1.23	0.62	0.08	0.94		0.11	0.52	0.46	1.23
D	0.89	0.57	0.22	0.04	0.34		0.12	0.40	0.92	1.62
E	0.56	0.68	0.23	0.02	0.21		0.06	0.10	1.08	1.03
pT	0.83	1.01	0.11	0.00	0.03		0.06	0.04	0.91	2.46
pY	1.10	1.63	0.28	0.07	0.12		0.56	0.77	1.16	1.61
